# No support for cryptic choice by ovarian fluid in an external fertilizer

**DOI:** 10.1002/ece3.4628

**Published:** 2018-11-08

**Authors:** Snøfrid April Kleppe, Jarle T. Nordeide, Geir Rudolfsen, Lars Figenschou, Berner Larsen, Katrin Reiss, Ivar Folstad

**Affiliations:** ^1^ Faculty of Biosciences and Aquaculture Nord University Bodø Norway; ^2^ Faculty of Bioscience, Fishery and Economy UiT The Arctic University of Norway Tromsø Norway; ^3^ University Library, UiT The Arctic University of Norway Tromsø Norway; ^4^ Nord University Business School Bodø Norway

**Keywords:** cryptic female choice, mate choice, ovarian fluid, *Salvelinus alpinus*, sperm competition

## Abstract

Whether the ovarian fluid (OF) represents a selective environment influencing cryptic female choice was tested using an external fertilizer experiencing intense sperm competition and large effects of OF on sperm swimming behavior—the Arctic charr (*Salvelinus alpinus*). We physically separated the OF from the eggs of reproductively active females and reintroduced either their own OF or fluid from another female to the eggs. The eggs were then fertilized in vitro in a replicated split‐brood design with sperm from two males under synchronized sperm competition trials, while also measuring sperm velocity of the individual males in the individual OFs. We found large effects of males, but no effect of females (i.e., eggs) on paternity, determined from microsatellites. More important, we found no effect of OF treatments on the relative paternity of the two competing males in each pair. This experimental setup does not provide support for the hypothesis that OF plays an important role as medium for cryptic female choice in charr. Power analyses revealed that our sample size is large enough to detect medium‐sized changes in relative paternity (medium‐sized effect sizes), but not large enough to detect small changes in relative paternity. More studies are needed before a conclusion can be drawn about OF's potential influence on paternity under sperm competition—even in charr.

## 1 INTRODUCTION

Sperm competition occurs when ejaculates from different males compete over fertilizing eggs either inside the females’ reproductive tract or externally to the female's body (Birkhead & Møller, 1998; Parker, 1970; Pizzari & Parker, 1998; Simmons, 2001; Stockley, Gage, Parker, & Møller, 1997). For external fertilizers, the outcome of this competition, that is, which male successfully sires the offspring, may be influenced by the relative competing abilities of the males’ ejaculates (sperm and seminal fluids), the female's spawning products (eggs and ovarian fluid [OF]), and their potential interactions. Thus, females of external fertilizers might not be regarded as only providing an arena for sperm competition, as they may also actively discriminate among which of the males involved in sperm competition is allowed to fertilize her eggs through cryptic choice (Birkhead, 1998; Eberhard, 1996; Olsson, Shine, Madsen, Gullberg, & Tegelstrom, 1996; Thornhill, 1983; Zeh & Zeh, 1996). Mechanisms used by females to control which male sires her eggs are expected to evolve in species with multiple matings—particularly in species that are exposed to intense sperm competition where female control of which males participate in the sperm competition is limited. This is the case for highly polyandrous broadcast spawners (Firman, Gasparini, Manier, & Pizzari, 2017), as for example in species with lek‐like mating systems. In some such species, cryptic female control under sperm competition has the potential to generate large differences in offspring survival (Rudolfsen, Figenschou, Folstad, Nordeide, & Søreng, 2005; Wedekind, Muller, & Spicher, 2001).

In several of the first studies of external fertilizers documenting that sperm velocity is of importance for fertilization success under sperm competition, sperm velocity was measured in water (e.g., Gage et al., 2004; Liljedal, Rudolfsen, & Folstad, 2008; Rudolfsen, Figenschou, Folstad, Tveiten, & Figenschou, 2006; Ottesen, Babiak, & Dahle, 2009; Skjæraasen et al., 2009). However, eggs are embedded in OF, which is a semiviscous liquid, sometimes comprising 10%–30% of the volume spawned (Lahnsteiner, 2002). The OF has several important effects on sperm traits including extending sperm longevity and increasing sperm velocity (Butts, Johnson, Wilson, & Pitcher, 2012; Golpour, Esfandyari, & Dadras, 2012; Lahnsteiner, 2002; Litvak & Trippel, 1998; Turner & Montgomerie, 2002; Urbach, Folstad, & Rudolfsen, 2005). More important, there is, at least in some species, an interaction effect of OF on sperm velocity (Rosengrave, Gemmell, Metcalf, McBride, & Montgomerie, 2008; Urbach et al., 2005). That is, OF of individual females specifically promote sperm velocity of certain males over others, suggesting that OF may be a medium in which females exert cryptic female choice (Beirão, Purchase, Wringe, & Fleming, 2015; Dietrich et al., 2008; Nordeide, 2007; Rosengrave et al., 2008; Urbach et al., 2005). Recent studies on external fertilizers have demonstrated that OF may reduce the velocity of sperm from subordinate males relative to that of dominant males (Egeland, Rudolfsen, Nordeide, & Folstad, 2016; Lehnert, Butts, et al., 2017; Makiguchi, Torao, Kojima, & Pitcher, 2016). Thus, subordinates, who invest more resources in their sperm and usually show the highest sperm velocity in water, have lower gains from their investments in sperm velocity than dominant males when sperm enter the OF surrounding eggs. Thus, females of external fertilizers may be promoting fertilizations by sperm from dominant males, not only by releasing their gonadal products close to and in synchrony with them, but also by promoting their sperm swimming performance in the immediate vicinity of the eggs.

Recent reviews and meta‐analysis have, however, provided weak evidence for genetic benefits from polyandry (Slatyer, Mautz, Backwell, & Jennions, 2012) and few clear demonstrations of cryptic female choice (Firman et al., 2017). Whereas some studies on external fertilizers have reported increased fertilization success for the male whose sperm swims faster in the females’ OF (e. g. Evans, Rosengrave, Gasparini, & Gemmell, 2013; Rosengrave, Montgomerie, & Gemmell, 2016; Lehnert, Butts, et al., 2017; Lehnert, Helou, Pitcher, Heath, & Heath, 2018), another study found no such relationship (Lehnert, Heath, Devlin, & Pitcher, 2017). Additionally, no effect of cryptic female choice on offspring fitness was documented in salmon (*Salmo salar*) reported by Lumley et al. (2016). Our knowledge about known proximate mechanisms enabling females to affect the outcome of sperm competition is limited (Firman et al., 2017). Important exceptions are the role of the sperm protein binding in egg–sperm recognition in sea urchins (Palumbi, 1999), and egg glycoproteins’ role in avoiding inbreeding in mice (Firman & Simmons, 2015). The role of MHC‐dependent gamete recognition might also be important in nonrandom gamete recognition and fusion in mice and salmonids (reviewed by Firman et al., 2017, Box 2). In Chinook salmon (*Oncorhynchus tshawytscha*), sperm from dominant males differ from those of subordinate males in flagellar beat frequency, bend length, bend angle, and wave amplitude when swimming in the OF (Butts, Prokopchuk, Kašpar, Cosson, & Pitcher, 2017). The fluid contains 174 proteins with individual variation in numbers and concentrations (Johnson et al., 2014) that may interact with the spermatozoa and modify their flagellar beating and velocity (Johnson et al., 2014; Rosengrave et al., 2016). Yet, although polyandrous and promiscuous females may derive fitness benefits from cryptic female choice, the general mechanisms are still unclear in the majority of taxa and species.

In external fertilizers like Salmonidae, it is not established whether the OF or the eggs themselves may influence the paternity during reproduction. Yet, the relative importance of these two factors might, at least in theory, nicely be identified by physically separating the OF from eggs of females and adding the OF from another female. These reproductive products could then be exposed to sperm competition trials, and the relative paternity from such trials could be compared to paternity when OF has not been exchanged between eggs from different females. Yeates et al. (2013) exchanged OF between eggs from salmon (*S. salar*) and brown trout (*S. trutta*) and reported that conspecific sperm gained fertilization precedence in interspecific sperm competition trials. Moreover, this precedence was primarily controlled by OF by increasing motility of conspecific sperm (Yeates et al., 2013). Yet, an intraspecific study on Chinook salmon (*O. tshawytscha*) documented no overall effect of OF on paternity success and no evidence for male–female interactions on paternity (Evans et al., 2013). This is surprising, as positive associations between sperm velocity in OF and both fertilization success and embryo survival have been reported in the same species (Rosengrave et al., 2016). Thus, the current experimental evidence suggests no intraspecific effect of OF on the outcome of sperm competition when exchanging OF between eggs from different females and exposing them to sperm competition.

Arctic charr (*Salvelinus alpinus*) is a good candidate for experimental repeats of Evans et al. (2013) study, since large male–female interaction effects on sperm velocity have been documented in OF from the species (Urbach et al., 2005). The charr is an external fertilizer showing large anisogamy where neither males nor females provide any form of parental care after spawning (Fabricius, 1953; Sørum, Figenschou, Rudolfsen, & Folstad, 2011). Throughout the one‐month‐long spawning season, males compete intensely over reproductive opportunities and demonstrate a lek‐like mating system at the easily observed spawning grounds (Figenschou, Folstad, & Liljedal, 2004; Liljedal & Folstad, 2003; Sigurjonsdottir & Gunnarsson, 1989; Sørum et al., 2011). Large dominant males behave aggressively toward smaller subordinate males (i.e., chase them away) when trying to guard the females from “sneakers” before spawning (Sigurjonsdottir & Gunnarsson, 1989). Yet, the spawning area provides no physical protection for the spawning pair and several males typically spawn in competition when the female releases her eggs (Sigurjonsdottir & Gunnarsson, 1989; Sørum et al., 2011). In our studied charr population, 76.5% of the ejaculates experience sperm competition and the mean number of males releasing milt in each spawning event is 2.6, suggesting a high level of sperm competition (Sørum et al., 2011). Sperm velocity and sperm density differ predictably between males adopting dominant or subordinate spawning strategies; that is, subordinates have more sperm with higher velocity in water, yet lower velocity in OF, than dominants (Egeland et al., 2016; Rudolfsen et al., 2006). Additionally, velocity of sperm in ejaculates has also been shown to influence fertilization success under sperm competition (Egeland, Rudolfsen, & JT, Folstad I., 2015; Liljedal et al., 2008).

In our present experiment, we conducted in vitro fertilization trials using charr gametes to disentangle the potential effects of eggs and OF in influencing relative paternity of two males under sperm competition. We first physically separated eggs and OF before embedding the eggs in either own or foreign OF. Thereafter, the eggs were fertilized by simultaneously releasing ejaculates from two males in competition, while also recording sperm speed in OF. If OF acts as a medium for cryptic female choice, we predicted that our experimental exchange of OF between eggs from the two females would influence paternity.

## 2 MATERIALS AND METHODS

### 2.1 Sampling and stripping of gametes

The charr were caught in Lake Fjellfrøsvatn located at 69°N, 19°E, at an altitude of 126 m in northern Norway, from the 18 to the 23 September in 2011 and 2012. Gillnets of 24 mm mesh size were used for fishing at three different spawning grounds (see Figenschou et al., 2004). Fish were removed from nets as soon as they were trapped to avoid injuries and thereafter stored in chicken wire cages by the shore until further handling. Males were caught less than 24 hr before they were stripped for gametes, whereas females—being more rare at the spawning grounds and hence more difficult to catch—were caught from 0 to 4 days prior to handling of the gametes. In the laboratory, the fish were put to death by a stroke to the head and fin tissue samples were obtained and kept in 70% ethanol for later genotyping. The area around the genital pore was then dried carefully by paper tissue in order to avoid contamination and subsequent activation of gametes, before the fish were stripped for free‐running gametes by a gentle bilateral pressure from the anterior part of the abdomen toward the genital pore. Handling of the gametes and fertilizations were carried out by the same experienced individuals on the 23 and 24 September in both years.

### 2.2 Experimental design

Two experiments were carried out in this study using a North Carolina II design. The first experiment (Sperm velocity analysis) was carried out to test whether the velocity of sperm from each of two males differed when swimming in each of two OF–water solutions from two different females. The details from this experiment are presented below (see Handling of male gametes). The second experiment (Paternity analysis) was performed to test for differences in relative paternity when sperm from two different males competed to fertilize eggs from a particular female which were surrounded by either its own OF or OF from another female. This second experiment was carried out in a block design where each block consisted of two males and two females that were tested in replicates (see Table 1). Females and males were randomly assigned to the blocks. In each trial, eggs of one female were treated either as “control,” “own OF,” or “foreign OF.” For each treatment, sperm of both males competed to fertilize the eggs in two replicates. We started out the experiment with eight full blocks, each including two trials as in Table 1, which resulted in 16 trials and the total sample size of 96 observations (in vitro fertilizations with 48 unique combinations of male‐egg donor‐ OF treatments, see below). However, in four of the blocks, one of the two females (i.e., one of the two trials in the block) produced eggs of poor quality and no eggs survived in one or more of the replicates. We therefore had to exclude one of the trials in each of these four blocks. Thus, our experiment consisted of four full blocks, each with 12 observations, and four half blocks, each with six observations. This gives a total of 72 observations (in vitro fertilizations with 36 unique combinations of male‐egg donor‐OF treatments, see below). A total of 649 offspring were genotyped, whereas the mean number of genotyped offspring in each the 72 fertilizations was 9.0. Since the fertilizations were carried out in two replicates (Table 1), the average total number of genotyped offspring per pair of parents per treatment (*control*,* own* and *foreign*) was 18.0 (minimum and maximum are 9 and 20 offspring, respectively).

**Table 1:** The experimental design used to test effects of ovarian fluid (OF) and sperm identity on fertilization success. For each block, two males and two females were used. In each trial, sperm from two males competed to fertilize eggs of one female which were treated as either “control” (untreated eggs), “own OF” (own ovarian fluid removed and added again, i.e., treatment control), or “foreign OF” (ovarian fluid removed and replaced with that of the other female). Each treatment combination per trial was replicated for a total of eight blocks. Due to eggs of poor quality, we had to exclude one of the two trials in four of the eight blocks. Thus, our experiment consists of 72 observations (in vitro fertilizations), with 36 unique “male ID–egg donor ID–ovarian fluid treatments” (observations or fertilizations). The offspring of each treatment combination was genotyped to assess paternity and counted in order to determine the relative paternity for each trial and treatment. The total number of offspring genotyped was 649, whereas the mean number of genotyped offspring in each of the 72 fertilizations was 9.0. Since the fertilizations were carried out in two replicates (Table 1), the mean total number of genotyped offspring per pair of parents per treatment (*control*,* own,* and *foreign*) was 18.0

Predictor	Block					
	Trial 1			Trial 2		
Male	Sperm ♂1 + sperm ♂2			Sperm ♂1 + Sperm ♂2		
Treatment	Control	Own OF	Foreign OF	Control	Own OF	Foreign OF
Egg‐donor ID	eggs ♀1 +	eggs ♀1	eggs ♀1 +	eggs ♀2 +	eggs ♀2	eggs ♀2
OF ID	OF ♀1	OF ♀1	OF ♀2	OF ♀2	OF ♀2	OF ♀1
Replicates	2	2	2	2	2	2

Fertilization success was measured as the ratio of offspring sired by the focal male (assigned to the male with the lower id number) to the sum of offspring of both males (termed “relative paternity”). “Relative sperm velocity” (i.e., “VCLdiff”) was measured as the difference in sperm velocity between the focal male and the competing male.

### 2.3 Handling of male gametes

All handling of gametes was conducted in a precooled laboratory. Immediately after stripping the milt from each male in a petri dish, we estimated milt volume (in 1‐ml syringes to the nearest 0.1 ml) and spermatocrit. When not handled, the milt was kept at 4°C in closed 1.5‐ml Eppendorf tubes and potential effects of handling time of sperm were minimized by conducting all the ejaculate measurements in the order by which the fish were included in the experiment. Sperm behavior was recorded 10 s postactivation, that is, as fast as possible. Sperm behavior was recorded first in lake‐water to ensure that the sperm were active, and then in water‐diluted OF (ratio of OF to water was 1 to 2, or 33% OF) of the two females in each block (see below). The same or similar dilution of water and OF was used in previous studies (e.g., Butts et al, 2012; Egeland et al., 2015; Egeland et al., 2016; Urbach et al., 2005). For simplicity, we hereafter refer to this mixture as OF. After placing less than 0.12 µl of sperm on a precooled (5–6°C) standard counting chamber (Leja Products BV), measurements were initiated after activating sperm by adding 4.5 µl of water or OF. To avoid possible effects of cell density on sperm behaviors, the slides were, immediately after sperm activation, screened for areas with an appropriate density of cells (average number of motile sperm was 97, *SD* = 42.4, *N* = 32). Records were made using a CCD black and white video camera module (Sony, XC‐ST50CE) attached to a CH30 Olympus microscope with a negative phase‐control objective lens (×10 magnification). All recordings were carried out in replicates. The video recordings were analyzed using an HTM‐CEROS sperm tracker (CEROS version 12; Hamilton Thorn Research, Beverly, MA), a standardized computer‐assisted sperm analysis (CASA) that has been shown to be an objective tool for measuring sperm characteristics (Elofsson, Van look, Borg, & Mayer, 2003; Kime et al., 1996; 2001; Rurangwa, Kime, Ollevier, & Nash, 2004). The image analyser was set as follows: frame rate 50 Hz; no. of frames 25; minimum contrast 11. To avoid including measurements taken of sperm cells moving due to drift or Brownian movement, threshold values for the only two optional settings defining static cells, that is, VAP and VSL, were set at 10 µm/s. The same method has successfully been used in previous studies (e.g., Janhunen et al., 2009; Liljedal et al., 2008; Urbach et al., 2005). The parameters assessed were average path velocity (VAP), straight‐line velocity (VSL), and curvilinear velocity (VCL) (Rurangwa et al., 2004). The data from the CASA revealed that these three sperm velocity parameters measured at 10 s after activation were significantly correlated, and Pearson's correlation coefficient was 0.96, 0.87, and 0.79 when comparing VCL and VAP, VSL and VAP, and VCL and VSL, respectively (*N* = 32 and *p* < 0.001 in each of the three correlations). Because of a lack of target (an egg) and no gradient in the concentrations of OF, we could not assume a straight‐line swimming behavior of sperm cells. Based on this, and the high correlation between the different velocity measurements (see above), we chose to use curvilinear velocity (from now on termed “sperm velocity” or “VCL”), as the measure of sperm velocity in our statistical models. Velocity estimates for each female–male (OF‐ejaculate) combination correspond to the mean velocity of all motile cells analyzed. We did not add any artificial substances, for example sperm extenders, to the milt.

### 2.4 Handling of female gametes

The stripped eggs embedded in OF from each female were stored in the dark at approximate lake water temperature until handling. In each block of the fertilization experiment (see Table 1), eggs from two females and OF from two females were treated in three different ways before sperm was added and eggs fertilized. We started by distributing eggs from each of the two females in each block into six batches (into six petri dishes) with three different treatments each with two replicates of similar egg numbers (similar egg numbers in each petri dish from one particular female, but the number varied between the females with minimum and maximum number of eggs per petri dish being 27 and 170, respectively) and OF volume. Within two of the six batches, the eggs were physically separated from the OF. The petri dish containing the eggs and OF was first tilted at approximately 30° long enough for the OF to drain from most of the eggs (1–2 min). The OF, now located at the lower part of the petri dish, was carefully removed with a pipette from the lowest point of the petri dish. This way of separating eggs and OF, by draining OF from the eggs, is probably similar to the draining of OF as reported by Lehnert et  al. (2018). Thereafter, the OF was returned to the same eggs (i.e., “own OF”). In the next two batches, the eggs and OF were separated as described above, but the OF was not returned to the same eggs; rather, the eggs were mixed with OF from the other female in the block (i.e., “foreign OF”). In the remaining two batches, the eggs and OF were not separated (i.e., “control”). The average volume of OF removed was 2.9 ml (*SD* = 1.58 ml), which gives approximately 0.5 ml OF per replicate. We did not wash the egg before swopping OF, nor did we pat the eggs dry or add any other artificial substances to the eggs or OF as we do not know the unforeseen effects of such treatment. The time elapsed from OF was removed from the eggs until it was re‐added or exchanged was approximately 45 min.

### 2.5 Fertilizations and rearing

Using micropipettes, milt from the two males was first added to the bottom of a glass jar carefully controlling for not allowing physical contact between the ejaculates. To ensure that differences in fertilization success between each of the two competing males in a pair were independent of initial difference in sperm numbers, the volume of the milt used in the sperm competition trials was adjusted according to the spermatocrit values to give an approximately equal number of sperm cells from each male. Fertilizations was conducted by adding 50 ml of lake water, mixing the two milt samples, and then gently pouring this mix over to a 500‐ml plastic jar already containing eggs embedded in OF. This was followed by 5 s gentle movement of the jar containing all gonadal products. The ratio of OF to water was approximately 1 to 100 during these first 5 s. Thereafter, the 500‐ml plastic jar was filled with water, in order to dilute sperm concentrations and avoid polyspermy, and sealed. Each of the batches was then stored separately in a refrigerator at 4°C–6°C until transportation to the hatchery, which occurred within 24 hr after fertilization. The eggs from each of the fertilizations were randomly positioned in two separate tanks and kept under a natural light regime in the hatchery at the University of Tromsø for the next 60 days (from fertilizations on 25 September in both 2011 and 2012) until they were killed with 75% ethanol when the experiment was terminated (the 15 November in both years). Untreated 6°C water was constantly exchanged in the tanks during this period.

### 2.6 Genotyping

Offspring and parents were genotyped using microsatellite DNA analysis. DNA extraction was done with the MasterPure^TM^ Complete DNA and RNA Purification Kit (EPICENTRE Biotechnologies, Cat. No. MC85200), following the instructions for tissue samples under point B user manual “Precipitation of Total Nucleic Acids and Precipitation of Total DNA.” When optimizing quantities of DNA, we used the Quant‐iT^TM^ dsDNA Broad‐Range Assay Kit (Invitrogen Detection Technologies, Lot. 1116429). Paternity was assigned using microsatellites and PCR. The PCR products (5 µl total volume) contained 200 ng DNA, 20 mM of primer forward, 20 mM primer reverse, and 1.25 units of AmpliTaq Gold 360 MM (Applied Biosystems, Life Technologies). The contents of each well were mixed by careful pipetting, no vortex. The plate was then sealed, spun shortly, and subjected to thermal cycling. PCR was performed with a Veriti^TM^ 96‐Well Thermal Cycler (Applied Biosystems, Life Technologies) by the following steps: Stage 1 (five cycles), 95°C 1:00 min, 55°C 0:20 min, 72°C 0:25 min; Stage 2 (five cycles), 95°C 0:30 min, 55°C 0:20 min, 72°C 0:25 min; Stage 3 (28 cycles), 95°C for 0:20 min, 55°C for 0:20 min, 72°C for 0:25 min; and Stage 4 (one time only), 72°C 20:00 min, 15°C for final temperature. The PCR products were then kept at 4°C until further handling. PCR products were separated on a 3500xl capillary sequencer (Applied Biosystems, Foster City, CA, USA), and alleles were identified by the ABI GeneMapper Software, version 4.1 (Applied Biosystems, Life Technologies). We used the following three microsatellite loci for genotyping: Smm_22 and Smm_24 (Crane et al., 2004), and Omm_1070 (Rexroad et al., 2001). In the present study, the number of unique alleles of Smm_22, Smm_24, and Omm_1070 of the 24 males and 24 females (parents) producing the 72 different fertilizations was 11, 10, and 4, respectively. The same microsatellites have also previously been reported as highly polymorphic in the present charr population (see Table 1 in Westgaard, Klemetsen, & Knudsen, 2004). The offspring were always identified as the offspring of one of the two fathers involved in each sperm competition, and one microsatellite was in most cases enough to unambiguously assign offspring to one father.

### 2.7 Statistical analyses

Statistical analyses were carried out similar to Evans et al. (2013) by using R version 3.3.2 (R Core Team, 2016). Two statistical analyses were conducted: sperm velocity analysis and paternity analysis. In the sperm velocity analysis, we tested potential effects of male identity and OF identity on sperm velocity (VCL). In this analysis, VCL was measured at 10 s after activation for the 32 different levels of the interaction variable between male identity and OF identity, and each measurement was replicated twice. Thus, the data set for the sperm velocity analysis contains 64 observations. We used linear mixed models (lmer) from the lme4 package (Bates, Maechler, Bolker, & Walker, 2016) including sperm velocity VCL (see above) as a continuous response variable and OF identity, male identity, and their interaction as random effects. Models with and without a specific random factor were compared using likelihood ratio tests from the R package lmtest (Zeileis & Hothorn, 2002), starting from the full model including the interactions.

In the paternity analysis, we tested the effects of both male identity and egg‐donor (i.e., the identity of the female's eggs as opposed to identity of the female's OF) on the paternity success of the focal male in the different fertilization treatments. The data set of this analysis consisted of 72 observations (i.e., in vitro fertilizations) with on average nine offspring, as described above. We used a binomial generalized linear mixed models (glmer from lme4) with a two‐column matrix as the response variable, where the first column consists of the numbers of offspring sired by the focal male, and the second column consists of the numbers of offspring sired by the second male. Note that this is equivalent to specifying relative paternity as the response variable together with specifying the parameter “weights” as the vector of the number of offspring sired by both males. Predictors were the treatments (“control,” “own OF,” and “foreign OF”) and relative sperm velocity (VCLdiff) as fixed effects, and male identity, egg‐donor, and their interaction as random effects. Note that egg‐donor and OF identity (used in sperm velocity analysis) are not in all cases the same as OF was exchanged in the “foreign OF” treatments (see Table 1). Starting from the full model, we stepwise simplified the model using likelihood ratio tests (see Sperm velocity analysis). We tested for overdispersion in the estimated model for the paternity data by using the overdispersion function in Bolker Ben and others (2017). There is no significant overdispersion in our model, as the overdispersion parameter and the *p*‐value were estimated to 1.15 and 0.19, respectively.

The study was carried out in accordance with ethical guidelines stated by the Norwegian Ministry of Agriculture and Food through the Animal Welfare Act. According to these guidelines, we were not supposed to—and therefore do not—have a specific approval or approval number.

## 3 RESULTS

### 3.1 Sperm velocity analysis

Sperm velocity in OF was significantly influenced by both male identity and OF identity (Table 2). That is, sperm from some males swam generally faster than sperm from other males in OF, and OF from some females affected sperm speed more than OF from other females. On the other hand, no interaction effect was revealed between OF identity and male identity (Table 2), suggesting that OF did not affect sperm velocity according to individual characteristics of ejaculates.

**Table 2:** Results from linear mixed models testing random effects of male identity (male ID), identity of ovarian fluid (OF ID), and their interaction, on the response variable sperm velocity in our data set of *N* = 64 observations. Sperm velocity was measured in ovarian fluid (OF) diluted in water. The standard deviations of the random effects were estimated by using the function lmer in the R package lme4, while the chi‐square statistics and the *p*‐values were measured by comparing models with and without this factor by likelihood ratio tests using the R function anova

Source	*SD*	Chi‐square	*p*‐Value
Male ID	14.69	17.104	<0.0001
OF ID	17.89	25.73	<0.0001
OF ID:male ID	5.56	0.395	0.53

### 3.2 Paternity analysis

**Figure 1:**
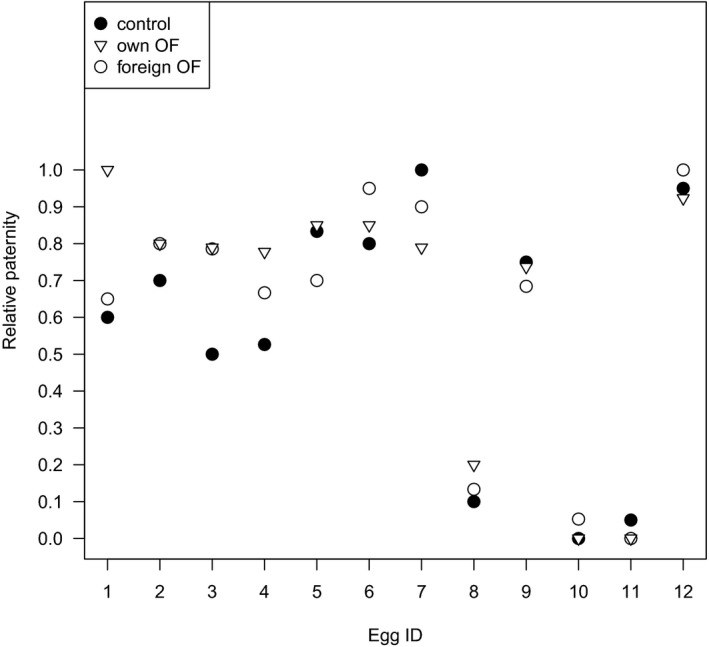
Scatter plot showing relative paternity of the focal male (offspring sired by the focal male/all offspring) in sperm competition trials including eggs from 12 different females. For each egg ID, relative paternity was measured in either “control” (●: untreated eggs), treatment “foreign OF” (○: own ovarian fluid removed and replaced with that of the other female in the block), or treatment “own OF” (▽: own ovarian fluid removed and back added, this is a treatment control). Each treatment combination per trial was replicated twice. The offspring of each in vitro fertilization was genotyped to assess paternity and counted in order to determine the relative paternity in each fertilization. Relative paternity is number of offspring sired by the focal male divided by the sum of offspring sired by both males in an in vitro fertilization. When computing the relative paternity, the number of offspring sired by the focal male for the two replications was added, and correspondingly for the number of offspring sired for by both males. Thus, each point in the scatter plot represents two replications

There was no support for OF affecting relative paternity between the two competing males. That is, relative paternity, measured as the ratio between the number of offspring sired by the focal male and the number of offspring sired by both males, was not influenced by our experimental exchange of OF between “egg batches” (see Table 3). Figure 1 illustrates that the relative paternity was not influenced by our experimental exchange of OF. Yet, relative paternity tended to differ between the treatments “control” versus “own OF” (*p* = 0.055, Table 3). However, this tendency was absent when comparing “own” versus “foreign OF” (*p* = 0.334) and when comparing “foreign” versus “control” (*p* = 0.348). Male identity significantly affected relative paternity (Table 3), but egg‐donor had no effect on paternity. Thus, although OF identity affected the sperm velocity (first analysis), there was no effect of any female parameter (egg‐donor or OF) on the relative paternity.

**Table 3:** Results from the generalized linear mixed models testing the effects of ovarian fluid (OF) on relative paternity. The response variable, relative paternity, was measured as number of offspring sired by the focal male divided by the sum of offspring sired by both males in each of the 72 in vitro fertilizations. Relative sperm velocity (i.e., the sperm velocity of the focal male minus sperm velocity of the competing male) was included as a continuous covariate. The treatments (“control,” “own OF,” and “foreign OF”) are dummy variables. In the table below, the last mentioned treatment on a line is the base value in this comparison. For example, “Treatment ‘control’ versus ‘own OF’” means that the effect of treatment “control” is estimated when treatment “own OF” is the base value. The test of “own versus control” tested for effects of removing the ovarian fluid and adding it back again, while “own versus foreign” tested for exchange of ovarian fluid. The chi‐square statistics and p‐values for the random factors were derived from likelihood ratio tests as in Table 2. All the other numbers in the table are output from the glmer function in the R package lme4

Source	Estimate	*SE*	*z*‐Value	*p*‐Value
Intercept	0.305	0.807	0.378	0.706
Treatment “control” versus “own OF”	−0.507	0.264	1.921	0.055
Treatment “own OF” versus “foreign OF”	0.266	0.275	0.966	0.334
Treatment “foreign OF” versus “control”	0.241	0.257	0.939	0.348
Relative sperm velocity	−0.006	0.004	−1.281	0.2

Source	Estimate	Var	Chi‐square	*p*‐Value
Egg‐donor ID		~0	~0	>0.999
Male ID		4.887	13.204	0.0003
Egg‐donor ID:male ID		~0	~0	>0.999

**Figure 2:**
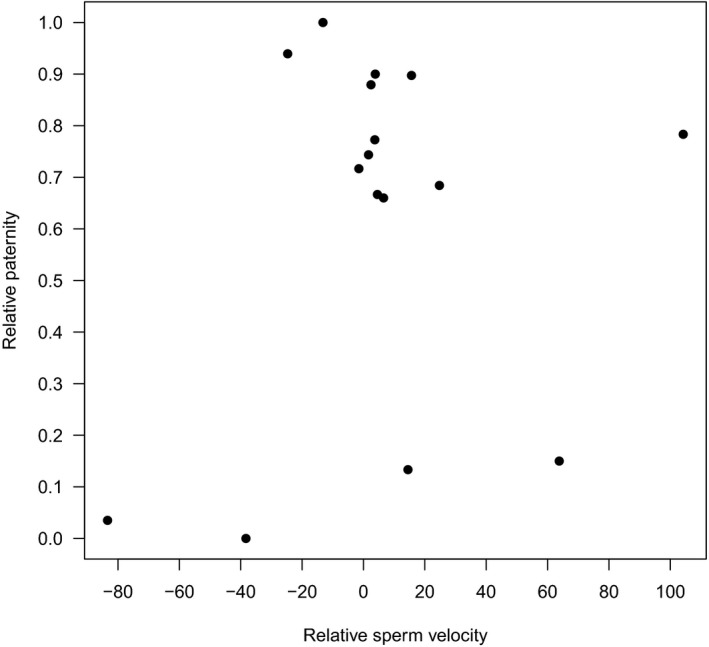
Scatter plot showing the relationship between relative paternity and relative sperm velocity under sperm competition. Relative sperm velocity is measured as the difference in sperm velocity of the focal male and the competing male for each of the two ovarian fluids in each of the eight blocks (see Table 1). Thus, there are in total 16 different values of relative sperm velocity in our data set. Relative paternity of these 16 different values of relative sperm velocity is the total number of offspring sired by the focal male in all observations with this value of relative sperm velocity, divided by the total number of offspring sired by both males in all observations with this value of relative sperm velocity. Note that the points represent different numbers of observations: six observations in the full blocks, and two or four observations in the half blocks

Relative velocity of the sperm from the two competing males did not affect relative paternity (Figure 2), suggesting that the observed difference in swimming speed between the two males in a pair was not important for fertilization success under our experimental setup.

### 3.3 Power analysis

As we did not find any significant fixed effects in the paternity analysis, we performed a power analysis by using the R package simr (Green & MacLeod, 2016). When using an effect size approximately at the level of the estimated coefficients (0.25 and 0.50, see “Estimate” in Table 3), the power of our tests of the influence of OF on relative paternity is indeed very low (0.07 and 0.18). However, statistical power is a function of the effect size, which in our experiment is the difference in relative paternity between the two males in sperm competition trials in OF from different females. The smaller the effect size, the smaller is the power. Effect sizes of 0.25 and 0.50 correspond to a change in relative paternity equal to 0.06 and 0.12, respectively, and Cohen's h equal to 0.12 and 0.24. Such effect sizes are classified as small according to Cohen (1988). On the other hand, an increase in the effect size (from 0.25 and 0.50 to*, i.e.,* 1.25) increases the power of our test of the influence of OF on relative paternity to 0.8, which is at an acceptable level. Such an effect size corresponds to a change in relative paternity between the two males in a trial equal to 0.25 and Cohen's *h* equal to 0.56, which according to Cohen is classified as a medium‐sized difference between proportions. So our sample size is large enough to detect medium‐sized changes in relative paternity, but not large enough to detect small changes in relative paternity. We have also computed the power of our test of the influence of relative sperm velocity on relative paternity. When using an effect size equal to −0.005, −0.01, and −0.025, the power of the test is 0.025, 0.115, and 0.76, respectively. Again, our test is not able to detect effect sizes at the level of the estimated coefficient −0.006 (see “Estimate” in Table 3) which is consistent with a p‐value of 0.2. However, the impact on relative paternity by such small effect sizes is very modest, partly due to the estimated coefficient being very small and partly due to the fact that most of the observed values of relative sperm velocities are in a small interval around 0, see Figure 1.

## 4 DISCUSSION

Our main finding is that exchange of OF between egg batches from different females did not affect the males’ relative fertilization success under sperm competition. Although sperm from some males generally swam faster in OF than sperm from other males, differences in sperm velocity between males had no effect on their relative fertilization success. Ovarian fluid from some females increased sperm speed more than OF from other females, but OF did not affect sperm velocity according to individual characteristics of ejaculates. Additionally, there was no indication that the eggs themselves favored sperm from one male over the other under sperm competition. Thus, the only two studies which have intraspecifically exchanged OF between eggs so far conclude that the effect size of cryptic choice exerted by OF is either small or absent (Evans et al., 2013; the present study). This result concurs to the main conclusion in a recent review, which found few clear demonstrations of cryptic female choice (Firman et al., 2017).

Common for experimental studies, our results may be affected by the applied methodology. First, the lack of effect when exchanging different OFs on paternity could result from our inability to remove all OF from the eggs. That is, we were not able to remove the last remains of the OF bound to the egg surface and the micropyle. On the other hand, we cannot exclude the possibility that physically removing the last remains of the original OF from the eggs by washing the eggs with water‐based isotonic fluid (Evans et al., 2013; Yeates et al., 2013) or by patting the eggs dry (Yeates et al., 2013) could affect the eggs and consequently the result of sperm competition. Evans et al. (2013) reported no effect of exchanging OF between females after washing the eggs with an artificial ovarian solution before adding foreign OF. Thus, the consistent results between our two studies—using slightly different methods on taxonomically closely related species—suggest that OF is not a medium exerting strong cryptic selection of sperm. It may be advocated that both methods applied to remove OF from the eggs are flawed, but this does not concur with the expected effect of OF on paternity documented interspecifically when eggs were also washed by isotonic solution (Yeates et al., 2013). It is therefore unlikely that different methods of separating OF from ova in the two studies caused the different results. Second, sperm velocity measurements were initiated 10 s after activation of sperm cells in the present study. Although as much as 80% of fertilizations in the river spawning sockeye salmon (*O. nerka*) may occur within 5 s (Hoysak & Liley, 2001), charr spawn in still water and show male–female interaction effects when sperm swim in OF as late as 30 s after activation. Our sampling delay is shorter or comparable to previous studies using model species that do not spawn in still water (Alonzo, Stiver, & Marsh‐Rollo, 2016; Evans et al., 2013; Yeates et al., 2013). Third, the concentration of OF (diluted in water) was 1% during the fertilizations in this study. Yeates et al. (2013) used the same (1%) concentration and reported significant effects of OF on paternity under interspecific sperm competition trials, whereas no effect of OF was revealed from intraspecific fertilizations at 10% solutions (Evans et al, 2013). Spawning behavior of charr in the present population has been studied by Sørum et al. (2011) and Brattli et al. (2018). The studies show that (a) more than 50% of the spawning events occur under sperm competition, (b) mean number of males is 2.9 s at egg release and increases to more than four males within the next 2.0 s, (c) the first male to release milt ejaculated from 0.15 s before to 1.9 s after the eggs are shed, and (d) the average time delay in gamete release under sperm competition between the first and the subsequent males is estimated as 0.68 s (Sørum et al., 2011). Moreover, some males have the advantage of spawning physically relatively close to the spawning female, whereas the remaining males spawn further away (Brattli, Egeland, Nordeide, & Folstad, 2018; Sørum et al., 2011). The exact concentration of OF diluted in water at the time of fertilizations in natural spawning events is not known for this population, but most likely it varies a lot between fertilizations and competing males. Thus, we cannot conclude whether 1% or 10% OF to water mimics the natural spawning conditions more closely. Fourth, a growth period from fertilization to sampling is needed for DNA‐sampling in order to estimate paternity. In the present study, this period lasted 60 days, that is, the same as that of Yeates et al. (2013), compared to 28 days in Evans et al. (2013). It seems inevitable that some eggs do not develop during this period as some eggs may be unfertilized and some zygotes malformed or dead. It is however unknown what causes specific mortality at this early stage. Yet, one possibility is that specific mortality differs because of varying “egg quality” due to physiological nonoptimal timing or other conditions during the artificial stripping and handling of gametes (Bobe & Labbé, 2010; Lahnsteiner, Weismann, & Patzner, 1999). An alternative explanation is that one of the two males in a block sired offspring with higher survival than the other male, that is, due to genetically superiority (Evans et al., 2013; García‐González, 2008). Under such “good‐sperm effects,” one would expect a positive and significant association within pairs of males between mortality of the eggs on the one hand and skewness in paternity on the other hand. A post hoc test carried out on the present data showed no association between relative paternity of the males and the proportion of eggs surviving (*r*_S_ = 0.134 *p* = 0.44, *N* = 36, Spearman's correlation coefficient after pooling both replicates). This result concurs with those previously reported from two independent studies using individuals from the same charr population, similar experimental designs, and the same rearing equipment and methods as in the present study (Egeland et al., 2015; Liljedal et al., 2008). It is therefore unlikely that the actual fertilization success we measure is caused by differential mortality or different developmental ability of the embryos sired by the two males in each block (see García‐González, 2008 for further discussion). Fifth, sperm velocity had no significant effect on relative paternity in our study (Table 3). The two males in each block were picked at random from the spawning grounds, and this lead to small within‐pair differences in sperm velocity. That is, relative sperm velocity differed by less than 10% in 26 of the 48 measurements (with “foreign OF,” “own OF,” and “control” combined). Such small between‐males differences in sperm velocity of our experimental pairs might explain why we did not find significant effects of relative sperm velocity on paternity. This is contrary to Egeland et al. (2015) who reported relative sperm velocity and motility as the best predictor of male fertilization success in charr from our study population. In the latter study, the two males whose sperm were competing to fertilize eggs in vitro were caged together for four days prior to fertilizations in order to deliberately produce one dominant male with low sperm velocity and one subordinate male with high sperm velocity (see also Egeland et al., 2016; Rudolfsen et al., 2006). This “production” of large differences in sperm velocity might explain the contrasting effects of sperm velocity on paternity in the two studies. In accordance with this explanation, Evans et al., (2013) who attributed chinook salmon males’ relative paternity to variation in the relative sperm competitive ability reported large variation in relative sperm velocity between the focal and the competing chinook males in their sperm competition experiment (fig. 3 in Evans et al., 2013). Sixth, a total of 649 offspring were genotyped in this study (see Materials and methods and Table 1), which is about 1/3 of the 1937 offspring genotyped by, for example, Evans et al., (2013). Moreover, our experiment consisted of four full blocks (one block consists of two trials) in addition to four trials (“half blocks”), which is approximately half the number of blocks (or trials) compared to Evans et al., (2013). The power analysis suggests that our sample size is large enough to detect medium‐sized effect sizes (changes in relative paternity from each male in the sperm competitions carried out in OF from two females), but not large enough to detect the small changes in relative paternity found in our study. Seventh, we picked the charr in each block by random and hence cannot exclude the possibility that the two females in each block (or trial) have very similar OF, for example, due to being closely related. If so, we should expect no effect on relative paternity. Eight, as the only study of this kind so far we included a second control group (“own OF,” see Materials and methods), to test for the potential effect of our handling of the female reproductive products by removing and then re‐adding the same OF to the same eggs. To our surprise, we found a large, but still nonsignificant, effect of this handling on paternity in the sperm competition trials. Future studies might explain this surprising result, and whether or notthe other potential problems discussed above have flawed the conclusion of the present study. Ninth, we do not have data on number of sperm:egg ratios in our fertilizations—see, for example, Butts, Trippel, and Litvak (2009) for such data in cod (*Gadus morhua*). Nor are we aware of studies which suggest appropriate sperm:egg ratios during fertilizations in Arctic charr. Thus, in this study—like in a number of other similar studies—one cannot exclude the possibility that the eggs were exhausted by too high numbers of sperm cells masking the potential ability of the OF to choose the best cells.

Sperm velocity was significantly associated with male identity and OF identity, but no interaction effect was found between the two in our study (see also Galvano, Johnson, Wilson, Pitcher, & Butts, 2013). Evans et al., (2013), on the other hand, reported the opposite result with significant interaction but no main effects. Two other studies on *S. alpinus* (Urbach et al., 2005) and *O. tshawytscha*; (Rosengrave et al., 2008) revealed significant effects of both males, OF, and their interaction. The inconsistency between studies is currently hard to explain, but may be related to different experimental designs, species‐specific effects, or some other unidentified variable. Additionally, micropylar sperm attractants at the egg surface within and immediately around the narrow micropylar opening leading to the egg interior have been suggested to attract or guide nearby spermatozoa toward and through the micropyle and may thus have the potential to affect sperm selection (Iwamatsu, Yoshizaki, & Shibata, 1997; Lehnert et al, 2018; Mengerink & Vacquier, 2001; Yanagimachi et al., 2013; Yanagimachi, Cherr, Pillai, & Baldwin, 1992). In fishes, glycoproteins are examples of such micropylar sperm attractants described (Iwamatsu et al., 1997; Yanagimachi et al., 1992). However, as we found no indication that the eggs themselves influenced relative paternity (see Table 3), there seem to be no evidence for sperm attractants influencing paternity in charr.

In conclusion, this study did not provide support for OF as a medium for cryptic female choice in charr under our experimental setup. Our data will contribute to future meta‐analyses on the potential effects of cryptic female choice in external fertilizers. More studies are needed before a conclusion can be drawn about OF's potential influence on paternity under sperm competition—even in charr.

## CONFLICT OF INTEREST

None declared.

## AUTHORS' CONTRIBUTION

SAK, JTN, GR, LF, and IF designed and performed the experiment. SAK and GR did the sperm analyses. SAK did the paternity analysis and IF reared the eggs. BL, KR, and JTN did the statistical analyses. All authors took part in the interpretation of the results and wrote parts of the manuscript, although most of the writings was carried out by SAK, JTN, and IF. All authors have read and approved the final version of the manuscript.

## DATA ACCESSIBILITY

The data used in the *Sperm velocity analysis* and *Paternity analysis* are archived on Dryad doi: https://doi.org/10.5061/dryad.7kd12bg.

## References

[ece34628-bib-0001] Alonzo, S. H. , Stiver, K. A. , & Marsh‐Rollo, S. E. (2016). Ovarian fluid allows directional cryptic female choice despite external fertilization. Nature Communications, 7, 12452 10.1038/ncomms12452. PMC4990696 27529581

[ece34628-bib-0002] Bates, D. , Maechler, M. , Bolker, B. , & Walker, S. (2016). lme4: Linear mixed‐effects models using Eigen and S4. R Packageversion 1.1‐12. Retrieved from https://cran.r-project.org/packages=lme4.

[ece34628-bib-0003] Beirão, J. , Purchase, C. F. , Wringe, B. F. , & Fleming, I. A. (2015). Inter‐population ovarian fluid variation differentially modulates sperm motility in Atlantic cod *Gadus morhua* . Journal of Fish Biology, 87, 54–68. 10.1111/jfb.12685. 25919195

[ece34628-bib-0004] Birkhead, T. R. (1998). Cryptic female choice: Criteria for establishing female sperm choice. Evolution, 52, 1212–1218. 10.1111/j.1558-5646.1998.tb01848.x 28565225

[ece34628-bib-0005] Birkhead, T. R. , & Møller, A. P. (1998). Sperm competition and sexual selection. San Diego, CA: Academic Press.

[ece34628-bib-0006] Bobe, J. , & Labbé, C. (2010). Egg and sperm quality in fish. General and Comparative Endocrinology, 165, 535–548. 10.1016/j.ygcen.2009.02.011. 19272390

[ece34628-bib-0007] Bolker Ben and others (2017). GLMM FAQ: Overdispersion. Retrieved from https://bbolker.github.io/mixedmodels-misc/glmmFAQ.html#testing-for-overdispersioncomputing-overdispersion-factor; factor

[ece34628-bib-0008] Brattli, M. B. , Egeland, T. B. , Nordeide, J. T. , & Folstad, I. (2018). Spawning behavior of Arctic charr (*Salvelinus alpinus*): Spawning synchrony, vibrational communication, and mate guarding. Ecology and Evolution, 8, 8076–8087. 10.1002/ece3.4277. 30250685 PMC6145006

[ece34628-bib-0009] Butts, I. A. E. , Johnson, K. , Wilson, C. C. , & Pitcher, T. E. (2012). Ovarian fluid enhances sperm velocity based on relatedness in lake trout, *Salvelinus namaycush* . Theriogenology, 78, 2105–2109.e2101. 10.1016/j.theriogenology.2012.06.031. 23110953

[ece34628-bib-0010] Butts, I. A. E. , Prokopchuk, G. , Kašpar, V. , Cosson, J. , & Pitcher, T. E. (2017). Ovarian fluid impacts flagellar beating and biochemical metrics of sperm between alternative reproductive tactics. Journal of Experimental Biology, 220, 2210–2217. 10.1242/jeb154195. 28615489

[ece34628-bib-0011] Butts, I. A. E. , Trippel, E. A. , & Litvak, M. K. (2009). The effect of sperm to egg ratio and gamete contact time on fertilization success in the Atlantic cod *Gadus morhua* L. Aquaculture, 286, 89–94. 10.1016/j.aquaculture.2008.09.005.

[ece34628-bib-0012] Cohen, J. (1988). Statistical power analysis for the behavioral sciences (2nd ed.). Lawrence: Erlbaum Associates.

[ece34628-bib-0013] R Core Team . (2016). R: A language and environment for statistical computing. Vienna, Austria: R Foundation for Statistical Computing https://www.R-project.org/.

[ece34628-bib-0014] Crane, P. A. , Lewis, C. J. , Kretschmer, E. J. , Miller, S. J. , Spearman, W. J. , DeCicco, A. L. , … Wenburg, J. K. (2004). Characterization and inheritance of seven microsatellite loci from Dolly Varden, *Salvelinus malma*, and cross‐species amplification in Arctic char, *S. Alpinus* . Conservation Genetics, 5, 737–741. 10.1007/s10592-004-1853-1

[ece34628-bib-0015] Dietrich, G. J. , Wojtczak, M. , Slowinska, M. , Dobosz, S. , Kuzminski, H. , & Ciereszko, A. (2008). Effects of ovarian fluid on motility characteristics of rainbow trout (*Oncorhynchus mykiss* Walbaum) spermatozoa. Journal of Applied Ichthyology, 24, 503–507. 10.1111/j.1439-0426.2006.01130.x.

[ece34628-bib-0016] Eberhard, W. G. (1996). Female control: Sexual selection by cryptic female choice. Princeton, NJ: Princeton University Press.

[ece34628-bib-0017] Egeland, T. B. , Rudolfsen, G. N. , Nordeide, J. T. , & Folstad, I. (2015). On the relative effect of spawning asynchrony, sperm quantity, and sperm quality on paternity under sperm competition in an external fertilizer. Frontiers in Ecology and Evolution, 3, 77 10.3389/fevo.2015.00077.

[ece34628-bib-0018] Egeland, T. B. , Rudolfsen, G. , Nordeide, J. T. , & Folstad, I. (2016). Status specific tailoring of sperm behavior in an external fertilizer. Frontiers in Ecology and Evolution, 4, 135 10.3389/fevo.2016.00135.

[ece34628-bib-0019] Elofsson, H. , Van look, K. , Borg, B. , & Mayer, I. (2003). Influence of salinity and ovarian fluid on sperm motility in the fifteen‐spined stickleback. Journal of Fish Biology, 63, 1429–1438. 10.1111/j.1095-8649.2003.00256.x

[ece34628-bib-0020] Evans, J. P. , Rosengrave, P. , Gasparini, C. , & Gemmell, N. J. (2013). Delineating the roles of males and females in sperm competition. Proceedings of the Royal Society of London. Series B: Biological Sciences, 280, 20132047 10.1098/rspb.2013.2047. 24266039 PMC3813331

[ece34628-bib-0021] Fabricius, E. (1953). Aquarium observations on the spawning behaviour of the charr, *Salmo alpinus* . Report: Institute of Fresh‐water Research, Drottningholm, 34, 14–48.

[ece34628-bib-0022] Figenschou, L. , Folstad, I. , & Liljedal, S. (2004). Lek fidelity of male Arctic charr. Canadian Journal of Zoology, 82, 1278–1284. 10.1139/z04-106

[ece34628-bib-0023] Firman, R. C. , Gasparini, C. , Manier, M. K. , & Pizzari, T. (2017). Postmating female control: 20 years of cryptic female choice. Trends in Ecology & Evolution, 32, 368–382. 10.1016/j.tree.2017.02.010 28318651 PMC5511330

[ece34628-bib-0024] Firman, R. C. , & Simmons, L. W. (2015). Gametic interactions promote inbreeding avoidance in house mice. Ecology Letters, 18, 937–943. 10.1111/ele.12471 26154782

[ece34628-bib-0025] Gage, M. J. G. , Macfarlane, C. P. , Yeates, S. , Ward, R. G. , Searle, J. B. , & Parker, G. A. (2004). Spermatozoal traits and sperm competition in Atlantic salmon: Relative sperm velocity is the primary determinant of fertilization success. Current Biology, 14, 44–47. 14711413

[ece34628-bib-0026] Galvano, P. M. , Johnson, K. , Wilson, C. C. , Pitcher, T. E. , & Butts, I. A. E. (2013). Ovarian fluid influences sperm performance in lake trout, *Salvelinus namaycush* Reproductive Biology, 13, 172–175. 10.1016/j.repbio.2013.02.001 23719125

[ece34628-bib-0027] García‐González, F. (2008). Male genetic quality and the inequality between paternity success and fertilization success: Consequences for studies of sperm competition and the evolution of polyandry. Evolution, 62, 1653–1665. 10.1111/j.1558-5646.2008.00362.x 18315573

[ece34628-bib-0028] Golpour, A. , Esfandyari, M. , & Dadras, H. (2012). The influence of ovarian fluid on the sperm physiology of *Rutilus kutum* . Iranian Journal of Fisheries Sciences, 14, 818–825.

[ece34628-bib-0029] Green, P. , & MacLeod, C. J. (2016). simr: An R package for power analysis of generalised linear mixed models by simulation. Methods in Ecology and Evolution, 7(4), 493–498. 10.1111/2041-210X.12504.

[ece34628-bib-0030] Hoysak, D. J. , & Liley, N. R. (2001). Fertilization dynamics in sockeye salmon and a comparison of sperm from alternative male phenotypes. Journal of Fish Biology, 58, 1286–1300. 10.1111/j.1095-8649.2001.tb02286.x

[ece34628-bib-0031] Iwamatsu, T. , Yoshizaki, N. , & Shibata, Y. (1997). Changes in the chorion and sperm entry into the micropyle during fertilization in the teleostean fish, *Oryzias latipes* . Development, Growth and Differentiation, 39, 33–41. 10.1046/j.1440-169X.1997.00005.x 9079033

[ece34628-bib-0032] Janhunen, M. , Rudolfsen, G. , Kekalainen, J. , Figenschou, L. , Peuhkuri, N. , & Kortet, R. (2009). Spawning coloration and sperm quality in a large lake population of Arctic charr (Salmonidae: *Salvelinus alpinus* L.). Biological Journal of the Linnean Society, 98, 794–802. 10.1111/j.1095-8312.2009.01317.x

[ece34628-bib-0033] Johnson, S. L. , Villaroel, M. , Rosengrave, P. , Came, A. , Kleffman, T. , Lokman, P. M. , & Gemmell, N. J. (2014). Proteomic analysis of chinook salmon (*Oncorhynchus tshawytscha*) ovarian fluid. PLoS ONE, 9, e104155 10.1371/journal.pone.0104155. 25089903 PMC4121310

[ece34628-bib-0034] Kime, D. E. , Ebrahimi, M. , Nysten, K. , Roelants, I. , Rurangwa, E. , Moore, H. D. M. , & Ollevier, F. (1996). Use of computer assisted sperm analysis (CASA) for monitoring the effects of pollution on sperm quality of fish; Application to the effects of heavy metals. Aquatic Toxicology, 36, 223–237. 10.1016/s0166-445x(96)00806-5.

[ece34628-bib-0035] Kime, D. E. , Van Look, K. J. W. , McAllister, B. G. , Huyskens, G. , Rurangwa, E. , & Ollevier, F. (2001). Computer‐assisted sperm analysis (CASA) as a tool for monitoring sperm quality in fish. Comparative Biochemistry and Physiology Part C: Toxicology & Pharmacology, 130, 425–433. 10.1016/S1532-0456(01)00270-8. 11738630

[ece34628-bib-0036] Lahnsteiner, F. (2002). The influence of ovarian fluid on the gamete physiology in the Salmonidae. Fish Physiology and Biochemistry, 27, 49–59. 10.1023/B:FISH.0000021792.97913.2e

[ece34628-bib-0037] Lahnsteiner, F. , Weismann, T. , & Patzner, R. A. (1999). Physiological and biochemical parameters for egg quality determination in lake trout, *Salmo trutta lacustris* . Fish Physiology and Biochemistry, 20, 375–388.

[ece34628-bib-0038] Lehnert, S. J. , Butts, I. A. E. , Flannery, E. W. , Peters, K. M. , Heath, D. D. , & Pitcher, T. E. (2017). Effects of ovarian fluid and genetic differences on sperm performance and fertilization success of alternative reproductive tactics in Chinook salmon. Journal of Evolutionary Biology, 30, 1236–1245. 10.1111/jeb.13088. 28387056

[ece34628-bib-0039] Lehnert, S. J. , Heath, D. D. , Devlin, R. H. , & Pitcher, T. E. (2017). Post‐spawning sexual selection in red and white Chinook salmon (*Oncorhynchus tshawytscha*). Behavioral Ecology, 28, 1–10.

[ece34628-bib-0040] Lehnert, S. J. , Helou, L. , Pitcher, T. E. , Heath, J. W. , & Heath, D. D. (2018). Sperm competition, but not major histocompatibility divergence, drives differential fertilization success between alternative reproductive tactics in Chinook salmon. Journal of Evolutionary Biology, 31, 88–97. 10.1111/jeb.13199. 29055057

[ece34628-bib-0041] Liljedal, S. , & Folstad, I. (2003). Milt quality, parasites, and immune function in dominant and subordinate Arctic charr. Canadian Journal of Zoology, 81, 221–227.

[ece34628-bib-0042] Liljedal, S. , Rudolfsen, G. , & Folstad, I. (2008). Factors predicting male fertilization success in an external fertilizer. Behavioral Ecology and Sociobiology, 62, 1805–1811. 10.1007/s00265-008-0609-1

[ece34628-bib-0043] Litvak, M. K. , & Trippel, E. A. (1998). Sperm motility patterns of Atlantic cod (*Gadus morhua*) in relation to salinity: Effects of ovarian fluid and egg presence. Canadian Journal of Zoology, 55, 1871–1877.

[ece34628-bib-0044] Lumley, A. J. , Diamond, S. E. , Einum, S. , Yeates, S. E. , Peruffo, D. , Emerson, B. C. , & Gage, M. J. G. (2016). Post‐copulatory opportunities for sperm competition and cryptic females choice provide no offspring fitness benefits in externally fertilizing salmon. Royal Society Open Science, 3, 150709. 27069665 10.1098/rsos.150709 PMC4821276

[ece34628-bib-0045] Makiguchi, Y. , Torao, M. , Kojima, T. , & Pitcher, T. E. (2016). Reproductive investment patterns and comparisons of sperm quality in the presence and absence of ovarian fluid in alternative reproductive tactics of masu salmon*, Oncorhynchus masou* . Theriogenology, 86, 2189–2192.e2. 27527407 10.1016/j.theriogenology.2016.07.009

[ece34628-bib-0046] Mengerink, K. J. , & Vacquier, V. D. (2001). Glycobiology of sperm – Egg interactions in deuterostomes. Glycobiology, 11, 37R–43R. 10.1093/glycob/11.4.37R 11358873

[ece34628-bib-0047] Nordeide, J. T. (2007). Is there more in 'gamete quality' than quality of the gametes? A review of effects of female mate choice and genetic compatibility on offspring quality. Aquaculture Research, 38, 1–16. 10.1111/j.1365-2109.2006.01635.x.

[ece34628-bib-0048] Olsson, M. , Shine, R. , Madsen, T. , Gullberg, A. , & Tegelstrom, H. (1996). Sperm selection by females. Nature, 383, 585–585. 10.1038/383585a0

[ece34628-bib-0049] Ottesen, O. H. , Babiak, I. , & Dahle, G. (2009). Sperm competition and fertilization success of Atlantic halibut (*Hippoglossus hippoglossus* L.). Aquaculture, 286, 240–245. 10.1016/j.aquaculture.2008.09.018

[ece34628-bib-0050] Palumbi, S. R. (1999). All males are not created equal: Fertility differences depend on gamete recognition mechanisms in sea urchins. Proceedings of the National Academy of Sciences, 96, 12632–12637. 10.1073/pnas.96.22.12632 PMC23023 10535974

[ece34628-bib-0051] Parker, G. A. (1970). Sperm competition and its evolutionary consequences in the insects. Biological Reviews, 45, 525–567. 10.1111/j.1469-185X.1970.tb01176.x

[ece34628-bib-0052] Pizzari, T. , & Parker, G. A. (1998). Sperm competition and sperm phenotype In Birkhead T. R., Hosken D., & Pitnick S. (Eds.), Sperm biology: An evolutionary perspective (pp. 207–245). Burlington, MA: Academic Press.

[ece34628-bib-0053] Rexroad, C. , Coleman, R. L. , Martin, A. M. , Hershberger, W. K. , & Killefer, J. (2001). Thirty‐five polymorphic microsatellite markers for rainbow trout (*Oncorhynchus mykiss*). Animal Genetics, 32, 317–319. 10.1046/j.1365-2052.2001.0730b.x 11683722

[ece34628-bib-0054] Rosengrave, P. , Gemmell, N. J. , Metcalf, V. , McBride, K. , & Montgomerie, R. (2008). A mechanism for cryptic female choice in chinook salmon. Behavioral Ecology, 19, 1179–1185. 10.1093/beheco/arn089.

[ece34628-bib-0055] Rosengrave, P. , Montgomerie, R. , & Gemmell, N. (2016). Cryptic female choice enhances fertilization success and embryo survival in chinook salmon. Proceedings of the Royal Society B, 283, 20160001 10.1098/rspb.2016.0001. 27009221 PMC4822462

[ece34628-bib-0056] Rudolfsen, G. , Figenschou, L. , Folstad, I. , Nordeide, J. T. , & Søreng, E. (2005). Potential fitness benefits from mate selection in the Atlantic cod (*Gadus morhua*). Journal of Evolutionary Biology, 18, 172–179. 10.1111/j.1420-9101.2004.00778.x 15669974

[ece34628-bib-0057] Rudolfsen, G. , Figenschou, L. , Folstad, I. , Tveiten, H. , & Figenschou, M. (2006). Rapid adjustments of sperm characteristics in relation to social status. Proceedings of the Royal Society of London, Series B: Biological Sciences, 273, 325–332. 10.1098/rspb.2005.3305. 16543175 PMC1560047

[ece34628-bib-0058] Rurangwa, E. , Kime, D. E. , Ollevier, F. , & Nash, J. P. (2004). The measurement of sperm motility and factors affecting sperm quality in cultured fish. Aquaculture, 234, 1–28. 10.1016/j.aquaculture.2003.12.006.

[ece34628-bib-0059] Sigurjonsdottir, H. , & Gunnarsson, K. (1989). Alternative mating tactics of Arctic charr, *Salvelinus alpinus*, in Thingvallavatn, Iceland. Environmental Biology of Fishes, 26, 159–176. 10.1007/BF00004814

[ece34628-bib-0060] Simmons, L. W. (2001). Sperm competition and its evolutionary consequences in the insects. Princeton, NJ: Princeton Univ. Press.

[ece34628-bib-0061] Skjæraasen, J. E. , Mayer, I. , Meager, J. J. , Rudolfsen, G. , Karlsen, Ø. , Haugland, T. , & Kleven, O. (2009). Sperm characteristics and competitive ability in farmed and wild cod. Marine Ecology Progress Series, 375, 219–228. 10.3354/meps07774.

[ece34628-bib-0062] Slatyer, R. A. , Mautz, B. S. , Backwell, P. R. Y. , & Jennions, M. D. (2012). Estimating genetic benefits of polyandry from experimental studies: A meta‐analysis. Biological Reviews, 87, 1–33. 10.1111/j.1469-185X.2011.00182.x 21545390

[ece34628-bib-0063] Sørum, V. , Figenschou, L. , Rudolfsen, G. , & Folstad, I. (2011). Spawning behaviour of Arctic charr (*Salvelinus alpinus*): Risk of sperm competition and timing of milt release for sneaker and dominant males. Behaviour, 148, 1157–1172. 10.1163/000579511x596615.

[ece34628-bib-0064] Stockley, P. , Gage, M. J. G. , Parker, G. A. , & Møller, A. P. (1997). Sperm competition in fishes: The evolution of testis size and ejaculate characteristics. American Naturalist, 149, 933–954. 10.1086/286031 18811256

[ece34628-bib-0065] Thornhill, R. (1983). Cryptic female choice and its implications in the scorpionfly *Harpobittacus nigriceps* . American Naturalist, 122, 765–788. 10.1086/284170

[ece34628-bib-0066] Turner, E. , & Montgomerie, R. (2002). Ovarian fluid enhances sperm movement in Arctic charr. Journal of Fish Biology, 60, 1570–1579. 10.1111/j.1095-8649.2002.tb02449.x

[ece34628-bib-0067] Urbach, D. , Folstad, I. , & Rudolfsen, G. (2005). Effects of ovarian fluid on sperm velocity in Arctic charr (*Salvelinus alpinus*). Behavioral Ecology and Sociobiology, 57, 438–444. 10.1007/s00265-004-0876-4

[ece34628-bib-0068] Wedekind, C. , Muller, R. , & Spicher, H. (2001). Potential genetic benefits of mate selection in whitefish. Journal of Evolutionary Biology, 14, 980–985. 10.1046/j.1420-9101.2001.00349.x

[ece34628-bib-0069] Westgaard, J. I. , Klemetsen, A. , & Knudsen, R. (2004). Genetic differences between two sympatric morphs of Arctic charr confirmed by microsatellite DNA. Journal of Fish Biology, 65, 1185–1191. 10.1111/j.0022-1112.2004.00524.x

[ece34628-bib-0070] Yanagimachi, R. , Cherr, G. , Matsubara, T. , Andoh, T. , Harumi, T. , Vines, C. , … Kaneshiro, K. (2013). Sperm attractant in the micropyle region of fish and insect eggs. Biology of Reproduction, 88(47), 1–11. 4710.1095/biolreprod.112.105072. 23303675 10.1095/biolreprod.112.105072

[ece34628-bib-0071] Yanagimachi, R. , Cherr, G. N. , Pillai, M. C. , & Baldwin, J. D. (1992). Factors controlling sperm entry into the micropyles of salmonid and herring eggs. Development, Growth & Differentiation, 34, 447–461. 10.1111/j.1440-169X.1992.00447.x 37281925

[ece34628-bib-0072] Yeates, S. E. , Diamond, S. E. , Einum, S. , Emerson, B. C. , Holt, W. V. , & Gage, M. J. G. (2013). Cryptic female choice of conspecific sperm controlled by the impact of ovarian fluid on sperm swimming behavior. Evolution, 67, 3523–3536. 10.1111/evo.12208. 24299405 PMC3912916

[ece34628-bib-0073] Zeh, J. A. , & Zeh, D. W. (1996). The evolution of polyandry I: Intragenomic conflict and genetic incompatibility. Proceedings of the Royal Society of London. Series B: Biological Sciences, 263, 1711–1717. 10.1098/rspb.1996.0250

[ece34628-bib-0074] Zeileis, A. , & Hothorn, T. (2002). Diagnostic checking in regression relationships. R News, 2(3), 7–10. https://CRAN.R-project.org/doc/Rnews/

